# Leveraging single-cell and multi-omics approaches to identify MTOR-centered deubiquitination signatures in esophageal cancer therapy

**DOI:** 10.3389/fimmu.2024.1490623

**Published:** 2024-12-17

**Authors:** Kang Tian, Ziang Yao, Da Pan

**Affiliations:** ^1^ Department of Oncology, The Affiliated Suqian Hospital of Xuzhou Medical University, Suqian, China; ^2^ Department of Traditional Chinese Medicine, Peking University People’s Hospital, Beijing, China; ^3^ Department of Gastroenterology, Wenzhou Central Hospital, Wenzhou, China

**Keywords:** esophageal squamous cell carcinoma, deubiquitination, TCGA, prognostic model, multi-omics

## Abstract

**Background:**

Esophageal squamous cell carcinoma (ESCC) remains a significant challenge in oncology due to its aggressive nature and heterogeneity. As one of the deadliest malignancies, ESCC research lags behind other cancer types. The balance between ubiquitination and deubiquitination processes plays a crucial role in cellular functions, with its disruption linked to various diseases, including cancer.

**Methods:**

Our study utilized diverse analytical approaches, encompassing Cox regression models, single-cell RNA sequencing, intercellular communication analysis, and Gene Ontology enrichment. We also conducted mutation profiling and explored potential immunotherapeutic agents. Furthermore, *in vitro* cellular experiments and *in vivo* mouse models were performed to validate findings. These methodologies aimed to establish deubiquitination-related gene signatures (DRGS) for predicting ESCC patient outcomes and response to immunotherapy.

**Results:**

By integrating datasets from TCGA-ESCC and GSE53624, we developed a DRGS model based on 14 deubiquitination-related genes (DUBGs). This signature effectively forecasts ESCC prognosis, drug responsiveness, and immune cell infiltration patterns. It also influences the mutational landscape of patients. Those classified as high-risk exhibited reduced survival rates, increased genetic alterations, and more complex cellular interactions, potentially explaining their poor outcomes. Notably, *in vitro* and *in vivo* experiments identified MTOR, a key component of the signature, as a promising therapeutic target for ESCC treatment.

**Conclusion:**

Our research highlights the significance of 14 DUBGs in ESCC progression. The risk score derived from this gene set enables clinical stratification of patients into distinct prognostic groups. Moreover, MTOR emerges as a potential target for personalized ESCC therapy, offering new avenues for treatment strategies.

## Introduction

Esophageal cancer (EC) is a common malignancy with a high mortality rate, affecting populations worldwide (1, 2). EC primarily includes two pathological classifications: esophageal adenocarcinoma and esophageal squamous cell carcinoma (ESCC), with ESCC accounting for approximately 90% of all cases (3). Despite significant advances in science and technology, the treatment of esophageal cancer remains challenging due to the high recurrence rate, limited molecular markers, and restricted therapeutic options (4, 5). The 5-year survival rate is disappointingly low, ranging from only 10% to 30% (6). Therefore, identifying specific molecular markers for the treatment and prognosis of ESCC is of critical importance (7–9).

Post-translational modification (PTM) is one of the key mechanisms for regulating various biological functions of cellular proteins (10). Different types of modifications can alter a protein’s charge, hydrophobicity, conformation, and stability, ultimately affecting its function. Among the most common PTMs are phosphorylation, acetylation, ubiquitination, glycosylation, and methylation (11, 12). Ubiquitination, in particular, has garnered significant attention due to its regulatory role in nearly all cellular processes, including the cell cycle, proliferation, apoptosis, differentiation, signal transduction, DNA repair, and immune and inflammatory responses (13, 14). Ubiquitin is a highly conserved polypeptide composed of 76 amino acids (8.5 kDa) that is widely expressed in eukaryotes (15). In the human genome, four genes encode ubiquitin proteins: UBB, UBC, UBA52, and RPS27A. The process of ubiquitination refers to the attachment of ubiquitin molecules to specific sites on substrate proteins, while deubiquitination is the removal of ubiquitin from substrate proteins by deubiquitinating enzymes (DUBs), counteracting ubiquitination (16). Ubiquitination and deubiquitination are crucial physiological processes related to the specific degradation of proteins and play a vital role in regulating cellular signaling pathways (17).

The functions of DUBs within the cell can be broadly categorized as follows: (1) Processing ubiquitin precursors to generate free ubiquitin molecules; (2) Removing ubiquitin chains from proteins to prevent their degradation by the proteasome, thereby stabilizing the proteins; (3) Detaching non-degradative ubiquitin signals from proteins; (4) Ensuring the stability of intracellular ubiquitin molecules by preventing their degradation alongside substrate proteins; (5) Participating in the disassembly of free ubiquitin chains within the cell; and (6) Editing the types of ubiquitin chains by cleaving them (18, 19).

DUBs play a crucial role in various cellular processes, including protein modification, localization, and the maintenance of cellular homeostasis, making them a highly promising therapeutic target (20). However, their pathogenic mechanisms and specific roles in disease progression, particularly in the development of ESCC, remain insufficiently understood and require further in-depth investigation. Therefore, exploring the relationship between DUBs, their associated pathways, and disease may lead to the discovery of new therapeutic targets and drugs, providing new insights into the treatment and prevention of diseases.

In this study, we integrated data from TCGA-ESCC and GSE53624 to develop reliable deubiquitination-related gene signatures (DRGS) capable of successfully predicting the survival of ESCC patients. Through a comprehensive analysis that included single-cell analysis, enrichment analysis, immune infiltration analysis, mutation analysis, and immune therapy drug inference, we explored the potential impact of DUBRGs on patient prognosis, immune cell infiltration, mutation landscape, and response to immunotherapy. Finally, we selected the key gene MTOR for *in vitro* and *in vivo* experimental validation. Our findings revealed that knockdown of the MTOR gene not only inhibited the growth of ESCC tumor cells *in vitro* but also reduced the growth rate of subcutaneous tumors in a mouse model, thereby improving the survival rate of the mice.

## Materials and methods

### Datasets and source

Gene expression RNAseq data and somatic mutation profiles in Mutation Annotation Format (MAF) were downloaded from The Cancer Genome Atlas (TCGA) database (https://portal.gdc.cancer.gov/). In the TCGA dataset, gene expression profiles were quantified using Transcripts Per Million (TPM) estimates and then log2-transformed for further analysis. GEO datasets were obtained from the Gene Expression Omnibus (GEO) of the National Center for Biotechnology Information (NCBI), which is publicly accessible at https://www.ncbi.nlm.nih.gov/geo.

### Establishment of DRGS for ESCC patients’ prognosis

After batch effects were removed from the TCGA-ESCC and GSE53624 datasets using the empirical Bayesian-based R package sva, we merged the data. Deubiquitination-related genes were identified as prognostic factors using univariate Cox regression, Lasso regression, and multivariate Cox regression analyses. Based on the results of the multivariate Cox regression, a mathematical formula was developed to predict the risk score for each ESCC patient:


$$Risk\;score=\sum_{i=1}^{n}\left(Exp_{i}\;*\;Coef_{i}\right)$$

Subsequently, all patients were divided into high-risk and low-risk groups according to the median risk score for further analysis.

### Differential expression and enrichment analysis

Based on gene expression profiles in ESCC, differential expression analysis was performed between high-risk and low-risk groups using the “*limma”* package in R to identify differentially expressed genes (DEGs). The thresholds for DEGs selection were defined as “adjusted P < 0.01 and |log(FoldChange)| > 0.5”. GO enrichment analysis was conducted using the “clusterProfiler” package in R, with a P-value less than 0.05 considered statistically significant. Data visualization was performed using the “ggplot2” package in R (21, 22). GO terms were categorized into three main ontology categories: Biological Process, Cellular Component, and Molecular Function.

### Immune analysis

Using multiple algorithms, including *CIBERSORT*, *MCPcounter*, *TIMER*, and *xCell*, we estimated the differences in the abundance of various immune cell types between high-risk and low-risk groups (23). These differences were visualized using a heatmap. Additionally, we performed a Spearman correlation analysis (r) to explore the relationship between the ESCC patient risk score and the StromalScore, ESTIMATEScore, ImmuneScore, and TumorPurity, with the results displayed in scatter plots.

### Mutation analysis

The R package *“Maftools”* was used to analyze and visualize the gene mutation profiles and frequencies in high-risk and low-risk groups, as well as to compare the differences in ESTIMATE scores, stromal scores, immune scores, and tumor purity between these groups (24). Tumor mutation burden (TMB) was calculated as the number of mutations per million bases (mut/Mb), and an oncoplot was generated using the *“oncoplot”* package to create a waterfall plot. Patients were divided into four groups based on TMB (high or low) and risk score (high or low), and Kaplan-Meier survival curves were plotted to assess the impact of TMB and risk score on overall patient survival.

### Single-cell RNA-sequencing analysis

Single-cell sequencing data for esophageal cancer was downloaded from the GEO database (GSE160269) and preprocessed using the *“Seurat-Req”* package (25) for single-cell RNA sequencing (scRNA-seq) analysis (26–28). The *“PercentageFeatureSet”* function was used to assess the proportion of mitochondrial genes within the dataset. To ensure data quality and completeness, only genes expressed in at least three cells were retained. The scRNA-seq data was normalized using the *“NormalizeData”* function. After normalization, the data was converted into a Seurat object, and the *“FindVariableFeatures”* function was used to identify the top 2,000 highly variable genes.

Subsequently, these highly variable genes were scaled and subjected to principal component analysis (PCA) using the *“RunPCA”* tool. Dimensionality reduction and visualization in two-dimensional space were achieved through the optimization of the Shared Nearest Neighbor (SNN) module and the t-distributed Stochastic Neighbor Embedding (t-SNE) clustering algorithm. Various cell subpopulations were annotated based on marker genes. We then analyzed the expression profiles of modeled DUBGs and classified the single-cell data into high- and low-risk groups based on DUBGs, evaluating the relationship between immune cell infiltration and risk scores.

Finally, *“CellChat”* was employed to assess differences in signaling patterns and interaction networks between cell types in the high- and low-risk groups (29, 30).

### Drug sensitivity analysis

Gene expression and drug sensitivity data for the same samples were downloaded from the *CellMiner* website (https://discover.nci.nih.gov/cellminer/). Drug sensitivity data were recorded after clinical laboratory validation and FDA standard certification. Pearson correlation analysis was used to determine the correlation between the risk score model and drug sensitivity.

### Cell culture

The human ESCC cell line (KYSE30) and the mouse ESCC cell line (mEC25) were obtained from the National Biomedical Experimental Cell Bank (Beijing, China) (31). The cells were cultured in 1640 medium (Gibco, USA)supplemented with 10% fetal bovine serum (Hyclone, USA)and 1% penicillin/streptomycin (Gibco, USA)in a humidified incubator with 5% CO_2_ at 37°C.

### Cell transfection

The day before transfection, plate the cells to ensure they reach 50-70% confluence at the time of transfection. On the day of transfection, prepare the transfection complex as follows: first, dilute the siRNA (sequences shown in **Table 1** for both human and mouse MTOR) and the transfection reagent separately in serum-free medium. Next, combine the diluted siRNA solution with the transfection reagent, gently mix, and incubate the mixture at room temperature for 15-20 minutes to allow the complex to form (GenePharma, Shanghai, China). Next, remove the culture medium from the cells, add the complex to the cells, and supplement with fresh culture medium. Incubate the cells at 37°C in a 5% CO_2_ incubator for 24-72 hours. To assess the efficiency of siRNA-mediated knockdown, total RNA was extracted from the transfected cells using a standard RNA isolation kit (e.g., TRIzol reagent) according to the manufacturer’s instructions. The RNA was then reverse-transcribed into cDNA using a reverse transcription kit. Quantitative real-time PCR (qPCR) was performed to measure the mRNA levels of the target gene (MTOR) using specific primers. The relative expression levels were normalized to GAPDH as an internal control, and knockdown efficiency was calculated using the 2^^(-ΔΔCt)^ method.

**Table 1:** The target sequences used for MTOR siRNA transfection are as follows:

Species	Number	Target sequence (5’-3’)	Ref Seq
Human	Sequence-1	CCGCTAGTAGGGAGGTTTATT	NM_004958
	Sequence-2	GCAACCCTTCTTTGACAACAT	NM_004958
Mouse	Sequence-1	GCTAGTTCGTATCAGCAGCAT	NM_020009
	Sequence-2	GCAGTGCTACACTACAAACAT	NM_020009

### CCK-8 assay

Transfected cells (5×10^3^) were seeded into the wells of a 96-well plate and incubated for 6 hours to allow adhesion (considered the starting point of the experiment, 0 h). Measurements were taken every 24 hours by adding 10 μL of CCK-8 reagent (MedChemExpress, USA) and 90 μL of 1640 medium to each well, followed by a 2-hour incubation at 37°C. The absorbance (OD) at 450 nm was then measured using a microplate reader (Thermo, USA). After a total of four measurements, a growth curve was plotted based on the OD values.

### Establishment of a subcutaneous tumor model in mice

The C57/BL6 mice used in the experiments were housed at the Xuzhou Medical University Animal Experiment Center. The animal ethics for this study were reviewed and approved by the Experimental Animal Ethics Committee of Xuzhou Medical University. All mice were kept in an SPF (Specific Pathogen-Free) environment with free access to food and water. All experimental procedures were conducted in strict accordance with ethical guidelines, ensuring that the design and execution of the experiments adhered to the highest standards of animal welfare and ethical considerations.

To establish a subcutaneous tumor model using the mEC25 mouse esophageal cancer cell line in 6-8 weeks-old C57BL/6 mice (SPF (Beijing)BIOTECHNOLOGY CO., LTD). Begin by culturing the mEC25 cells until they reach the logarithmic growth phase. Harvest the cells and resuspend them in sterile PBS at a concentration of 1x10^7 cells/ml. Using an insulin syringe, inject 100 µL of the cell suspension subcutaneously into the right flanks of each mouse. Every 2-3 days, measure the tumor’s length (L) and width (W) using calipers, and calculate the tumor volume using the formula V = (L x W^2^)/2.

### Statistical analysis

Data analysis and visualization were conducted using R version 4.0.2. Categorical variables were compared using the chi-square test, while group differences were assessed using the Student’ t-test and the Wilcoxon rank-sum test. The correlation between two parameters was evaluated using Spearman correlation analysis. All statistical tests were two-sided, and a P-value of less than 0.05 was considered statistically significant.

## Result

### Construction and validation of a prognostic model related to deubiquitination

**Figure 1:**
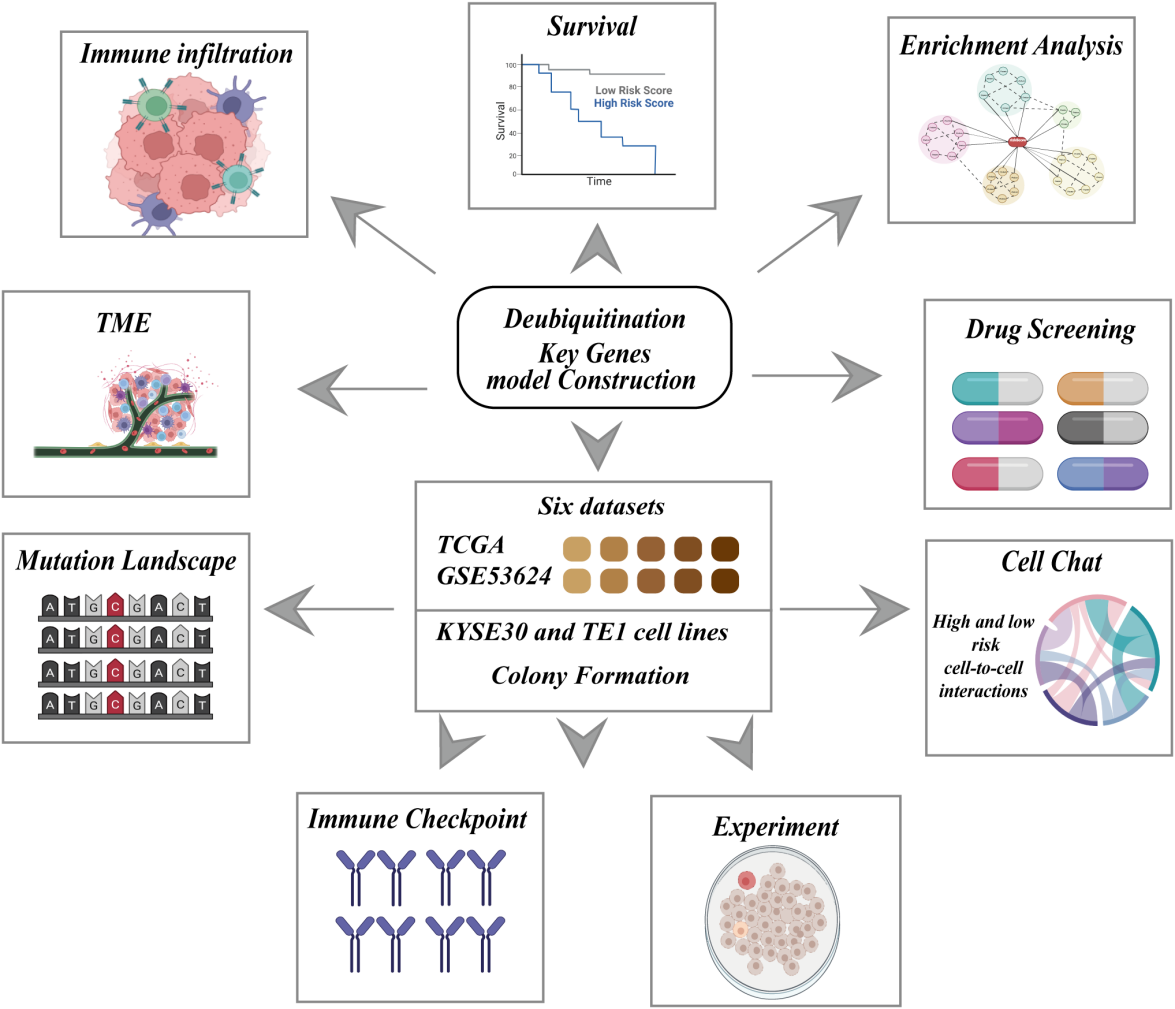
Schematic representation of the research framework for constructing a deubiquitination related key genes model. The central block highlights the integration of six datasets, including TCGA and GSE53624, as well as experiments using KYSE30 and TE1 cell lines, and colony formation assays. This comprehensive approach is employed to construct a deubiquitination key genes model, which is subsequently evaluated through multiple analyses.

**Figure 2:**
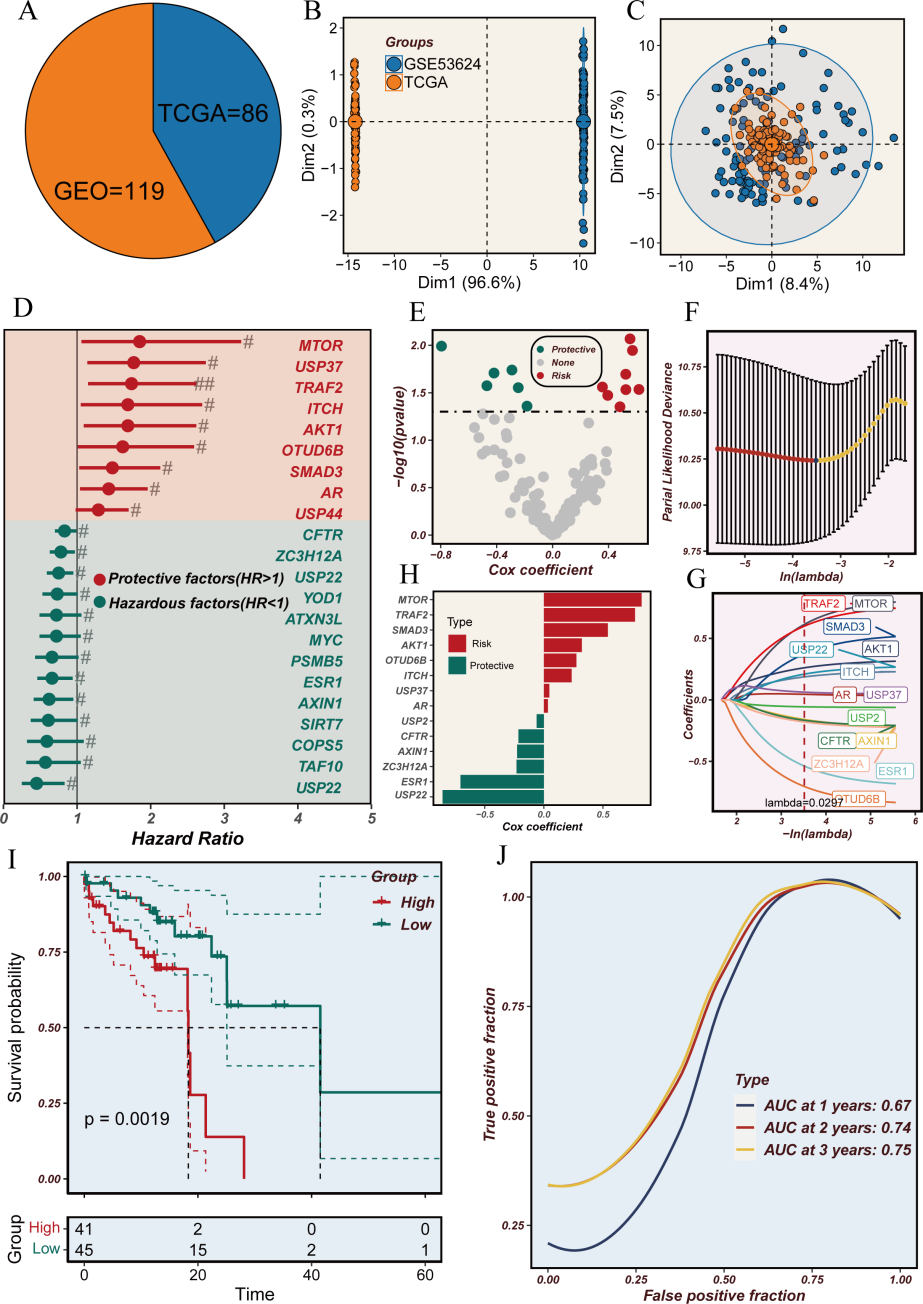
Analysis of deubiquitination-related genes in the TCGA and GEO cohorts. **(A)** Pie chart representing the distribution of samples from TCGA (n=86) and GEO (n=119) cohorts. **(B)** Principal component analysis (PCA) plot showing the variance between samples from TCGA and GEO datasets. **(C)** PCA plot illustrating the clustering of samples from TCGA and GEO datasets. **(D)** Forest plot showing the hazard ratios (HR) for deubiquitination-related genes. Genes with HR > 1 are considered hazardous factors, and those with HR < 1 are considered protective factors. **(E)** Volcano plot of Cox regression coefficients for deubiquitination-related genes, highlighting protective and hazardous factors. **(F)** Partial likelihood deviance plot for LASSO regression analysis to determine the optimal lambda value. **(G)** LASSO coefficient profiles of deubiquitination-related genes with varying lambda values. **(H)** Bar plot of Cox coefficients, distinguishing risk and protective factors among deubiquitination-related genes. **(I)** Kaplan-Meier survival curves comparing high-risk and low-risk groups based on deubiquitination-related gene expression. **(J)** Time-dependent ROC curves showing the predictive accuracy (AUC) at 1, 2, and 3 years.

The complete workflow is shown in **Figure 1**. To explore the potential of utilizing DRGS for clinical decision support in ESCC, we combined the TCGA-ESCC (n=86) and GSE53624 (n=119) datasets to develop a prognostic model for ESCC. Initially, batch effects between the two datasets were removed (**Figures 2A–C**), followed by univariate Cox analysis to identify DUBs significantly associated with overall survival (OS) in ESCC patients (**Figure 2D**). To address overfitting risks and refine gene selection for OS prediction, we performed LASSO regression analysis, and stepwise multivariate Cox analysis subsequently identified USP2, ITCH, ESR1, AXIN1, MTOR, USP22, TRAF2, USP37, AKT1, AR, OTUD6B, ZC3H12A, and SMAD3 as independent prognostic factors. These genes were used to construct the DRGS for ESCC patients. The risk score was calculated by summing the expression levels of individual genes, each weighted by their respective regression coefficients (**Figure 2H**). Patients were then divided into high- and low-risk groups based on the median risk score (**Figures 2E–G**). Kaplan-Meier survival analysis revealed that ESCC patients in the high-risk group had significantly shorter survival probabilities, with 1-, 3-, and 5-year AUCs of 0.67, 0.74, and 0.75, respectively, indicating a robust predictive capability of the DRGS for ESCC patients (**Figures 2I–J**).

### Exploration of mutational landscapes and biological mechanisms

**Figure 3:**
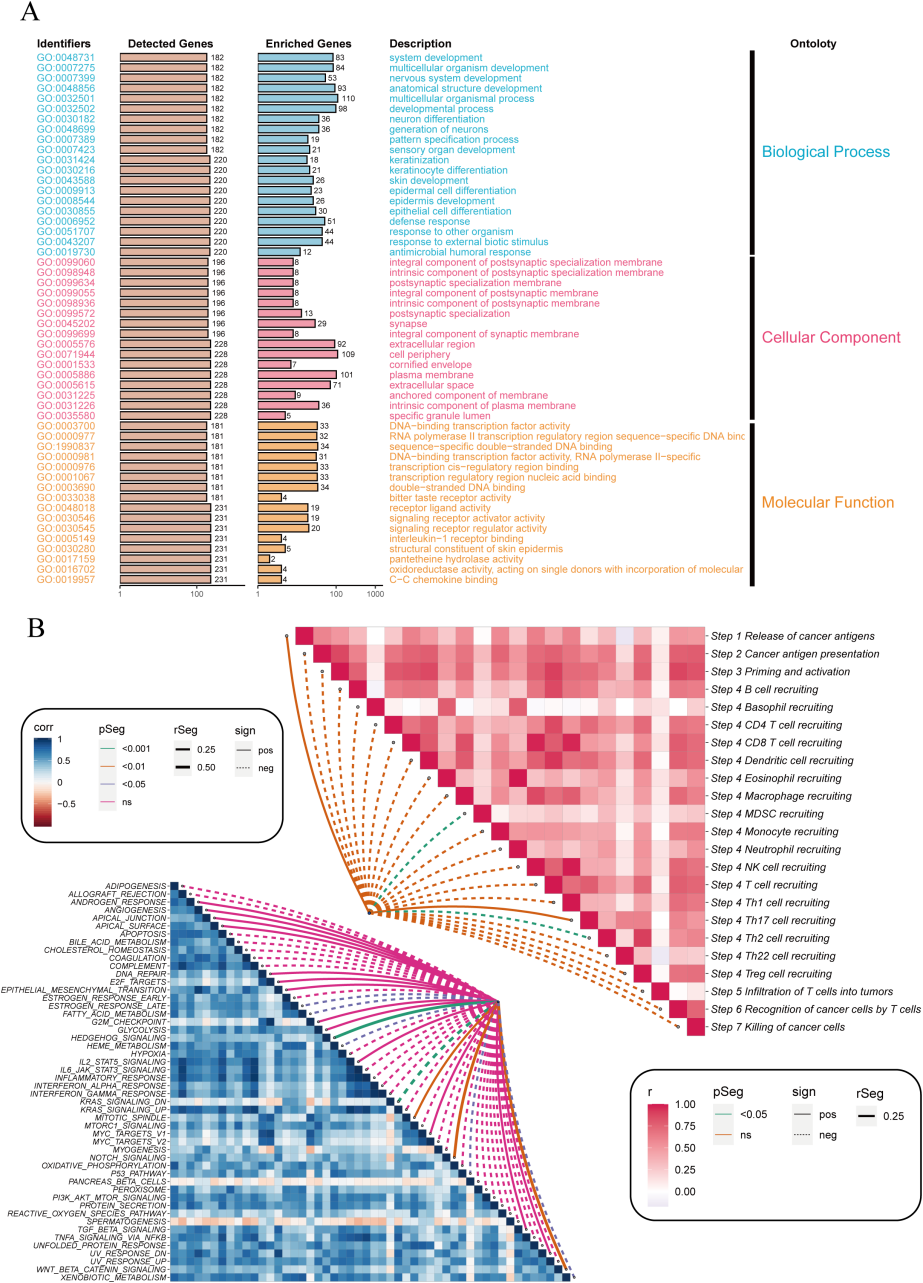
Enrichment analysis of differentially expressed genes between high and low-risk groups. **(A)** Bar plot showing the Gene Ontology (GO) enrichment analysis for differentially expressed genes. The identified GO terms are categorized into three main ontologies: Biological Process (blue), Cellular Component (orange), and Molecular Function (pink). The y-axis lists the GO identifiers, and the x-axis shows the number of detected and enriched genes for each GO term. **(B)** Correlation heatmap illustrating the relationship between immune-related pathways (right) and cancer-related pathways (left). The color intensity represents the correlation coefficient (r), with red indicating positive correlations and blue indicating negative correlations. The dotted lines connect pathways with significant correlations, highlighting key interactions involved in cancer immunity. NS stands for "Not Significant".

Next, we explored the functional classification of DRGS in ESCC patients and their potential roles and impacts in cancer immunity. As shown in **Figure 3A**, the Gene Ontology (GO) analysis indicates that DRGS may influence ESCC progression by participating in the regulation of biological processes such as Pattern Specification Process, Epidermal Cell Differentiation, Epidermis Cell Differentiation, Response to Other Organism, Response to External Biotic Stimulus, and Antimicrobial Humoral Response, thereby affecting patient prognosis. **Figure 3B** also suggests that DRGS may be involved in processes such as the release of cancer antigens, the presentation and activation of cancer antigens, immune cell recruitment, and tumor killing.

**Figure 4:**
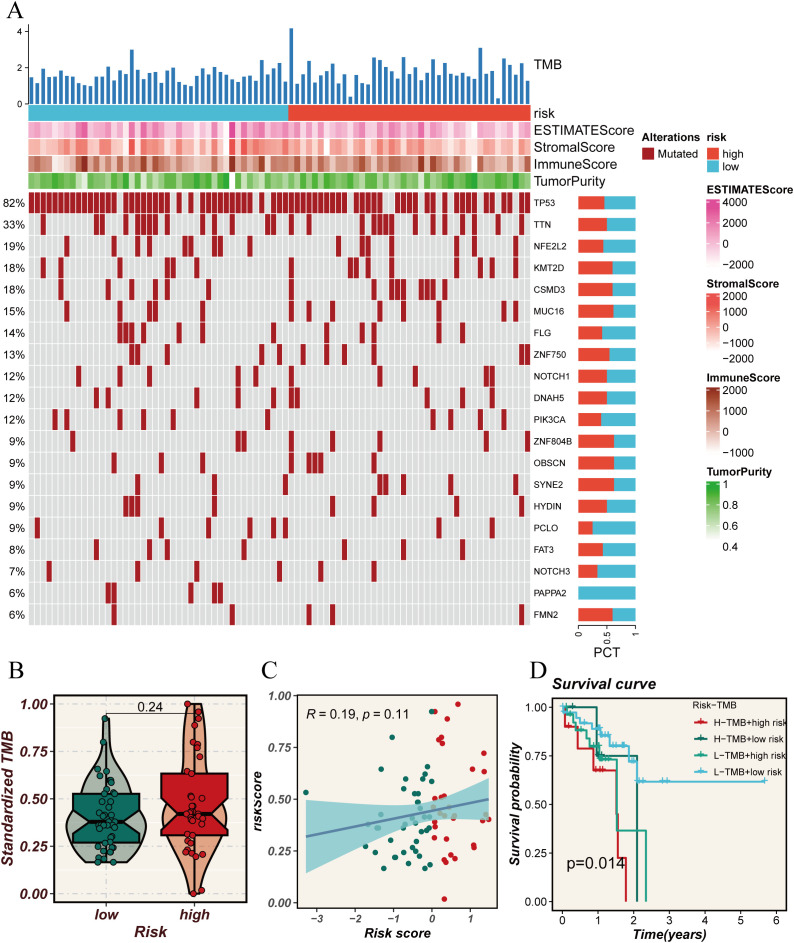
Analysis of tumor mutational burden (TMB) in relation to risk scores. **(A)** Oncoprint plot showing the mutation landscape across high and low-risk groups. The top bar plot represents the TMB, while the heatmaps illustrate the risk scores, ESTIMATE scores, Stromal scores, Immune scores, and tumor purity for each sample. The percentage on the left indicates the frequency of mutations in each gene across the cohort. **(B)** Violin plot comparing the standardized TMB between high and low-risk groups. **(C)** Scatter plot showing the correlation between risk scores and standardized TMB. The blue line represents the regression line, with the shaded area indicating the confidence interval. **(D)** Kaplan-Meier survival curves comparing overall survival across four groups categorized by high and low TMB and risk scores. The p-value indicates the statistical significance of the differences between the groups.

To explore the molecular mechanisms driving the abnormal expression of these 14 DUBGs, we examined the differences in TMB between high-risk and low-risk groups of ESCC patients. The results indicate that the high-risk group has a higher mutation frequency in several genes (such as TP53, TTN, NFE2L2) compared to the low-risk group. Additionally, the high-risk group exhibits generally lower ESTIMATE Score, Stromal Score, and Immune Score (**Figure 4A**). The high-risk group also has a higher standardized Tumor Mutation Burden (TMB, **Figure 4B**), and there is a positive correlation between the risk score and TMB, though not statistically significant (**Figure 4C**). The combination of TMB and risk score significantly influences patient survival, with the combination of low TMB and low risk being associated with better survival outcomes (**Figure 4D**).

### DUBGs predicts immune cell infiltration of ESCC patients

**Figure 5:**
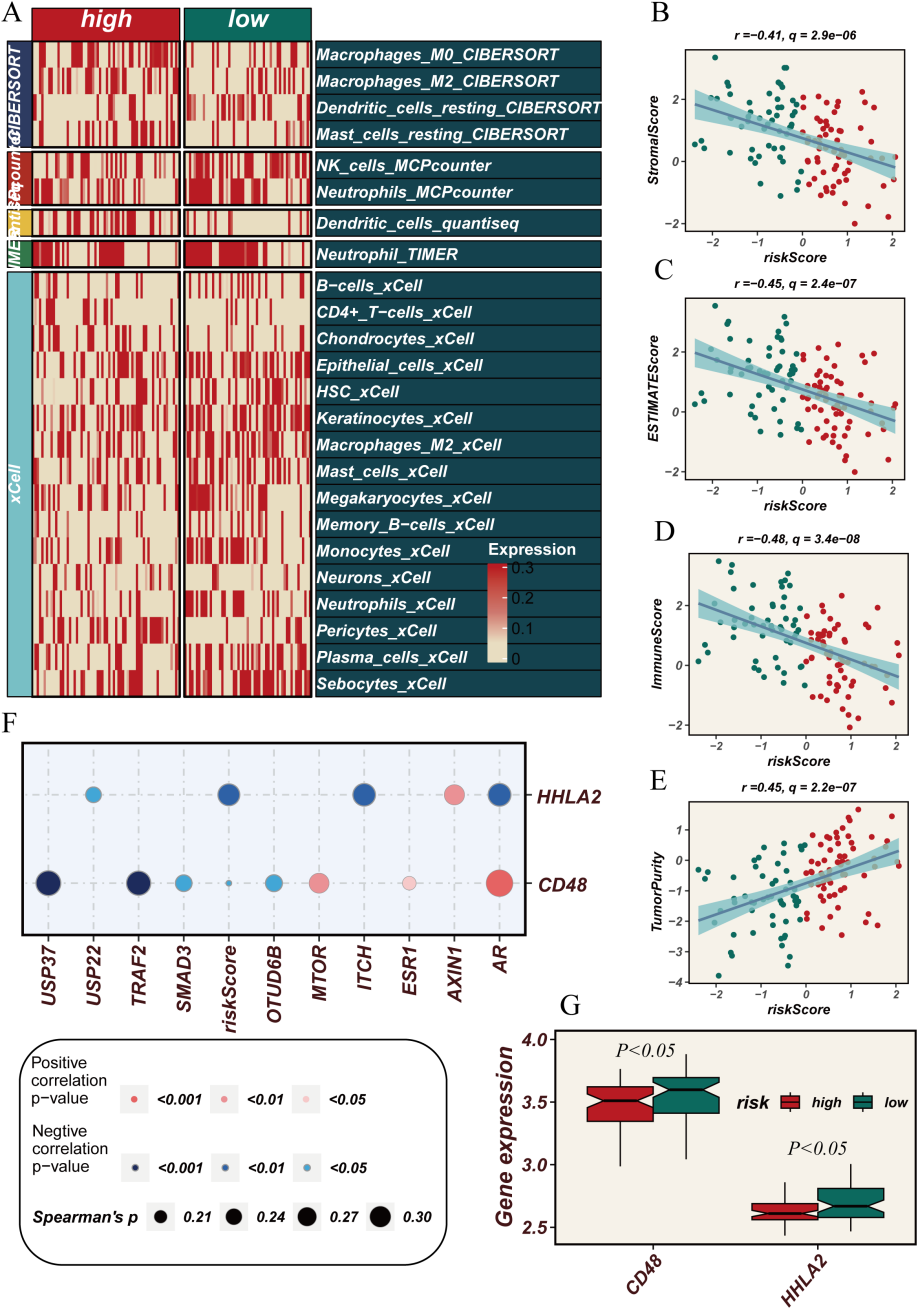
Correlation between immune cell infiltration, tumor microenvironment scores and risk scores. **(A)** Heatmap showing the comparison of immune cell infiltration levels between high and low-risk groups across various immune cell types as quantified by multiple algorithms (CIBERSORT, MCPcounter, xCell, etc.). **(B–D)** Scatter plots showing the negative correlation between risk scores and **(B)** StromalScore, **(C)** ESTIMATEScore, and **(D)** ImmuneScore. **(E)** Scatter plot showing the positive correlation between risk scores and tumor purity. **(F)** Dot plot showing the correlation between the expression of immune-related genes (HHLA2, CD48) and deubiquitination-related genes with risk scores. **(G)** Box plots comparing the expression levels of CD48 and HHLA2 between high and low-risk groups, showing a statistically significant difference (P < 0.05).

The previous analyses suggest that DUBGs may influence immune cell infiltration in ESCC patients. Therefore, we conducted a more detailed investigation to explore the relationship between risk scores and immune cell infiltration, immune-related gene expression, and tumor purity in ESCC patients. Using different computational methods such as CIBERSORT, MCPcounter, and xCell, we observed that the expression levels of various immune cells, including Macrophages_M0, NK_cells, Dendritic_cells, B-cells, CD4+ T-cells, Memory_B-cells, and Monocytes, were lower in the high-risk group compared to the low-risk group, particularly in the xCell analysis (**Figure 5A**). Additionally, correlation analysis revealed that risk scores were negatively correlated with Stromal Score, ESTIMATE Score, and Immune Score, but positively correlated with Tumor Purity, indicating that higher risk scores are associated with higher tumor purity (**Figures 5B–E**). Moreover, key genes such as USP37, USP22, TRAF2, SMAD3, and MTOR showed a positive correlation with immune-related genes HHLA2 and CD48 (**Figure 5F**), and the expression levels of HHLA2 and CD48 were significantly higher in the high-risk group (p < 0.05, **Figure 5G**). These analysis underscores the potential role of DUBGs in modulating immune cell infiltration and highlights the relationship between risk scores, immune gene expression, and tumor microenvironment characteristics in ESCC patients.

### Single-cell sequencing reveals the potential biological impact of DUBGs on ESCC patients

**Figure 6:**
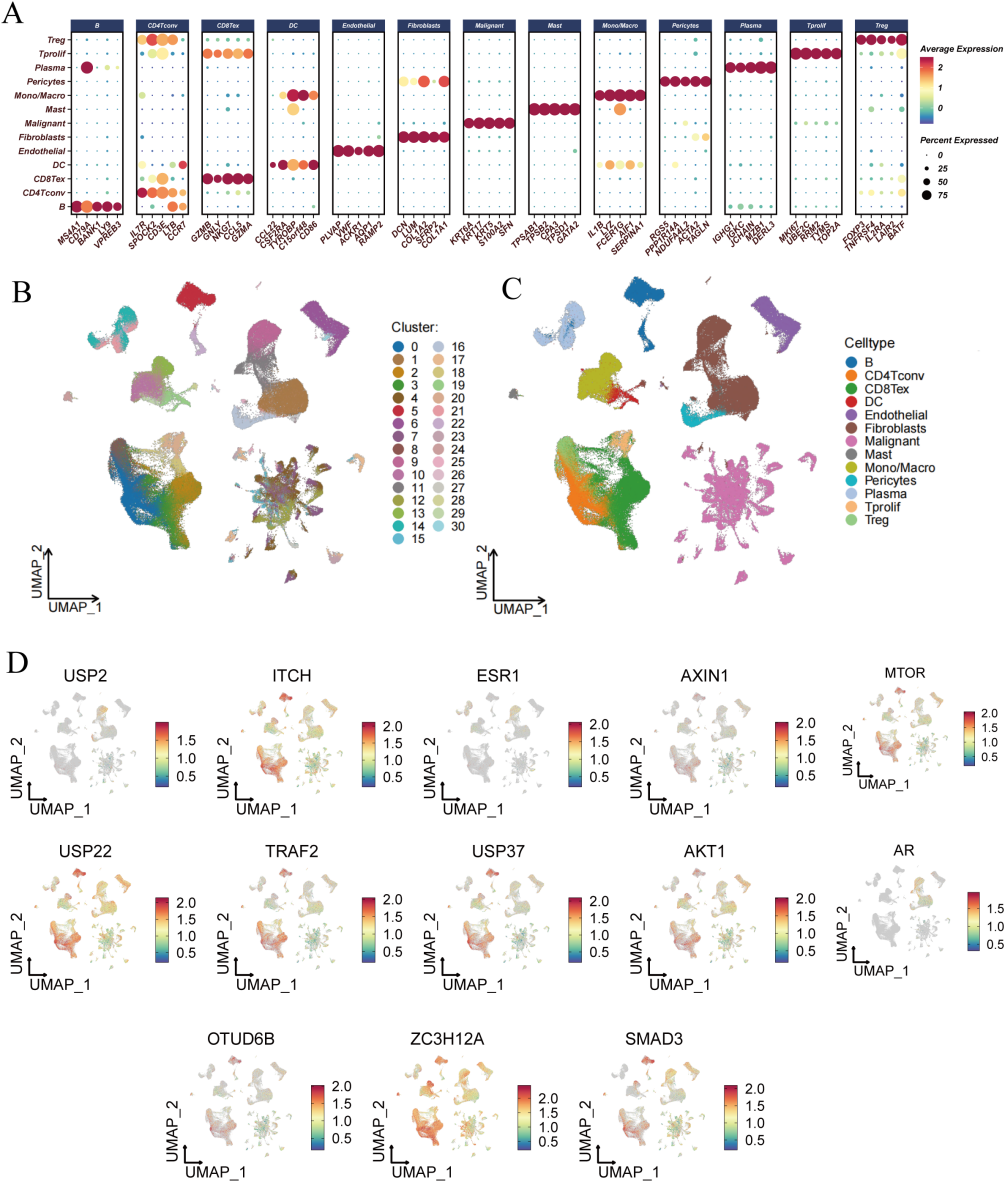
Single-cell RNA-seq analysis and risk score evaluation of deubiquitination-related genes across different cell types. **(A)** Dot plot showing the expression levels of deubiquitination-related genes across various cell types, including B cells, CD4+ T cells, CD8+ T cells, DCs, Endothelial cells, Fibroblasts, Malignant cells, Mast cells, Monocytes/Macrophages, Pericytes, Plasma cells, Tprolif, Tregs, and others. **(B)** UMAP plot showing the clustering of different cell types based on their gene expression profiles. Each cluster is color-coded and numbered. **(C)** UMAP plot highlighting the distribution of specific cell types, such as B cells, T cells, DCs, Endothelial cells, and others, within the entire dataset. **(D)** UMAP plots illustrating the expression patterns of individual deubiquitination-related genes (e.g., USP2, ITCH, ESR1, etc.) across different cell clusters.

To gain a deeper understanding of the underlying mechanisms by which DUBGs influence the function and gene regulation in esophageal cancer cells, we conducted a single-cell level analysis. We first performed dimensionality reduction, clustering, and cell identification on the single-cell sequencing data of esophageal cancer from the GSE160269 dataset. A total of 13 cell types were identified: B cells, CD4Tconv cells, CD8Tex cells, DC cells, Endothelial cells, Fibroblasts, Malignant cells, Mast cells, Mono/Macro cells, Pericytes, Plasma cells, T prolif cells, and Treg cells (**Figures 6A–C**).

**Figure 7:**
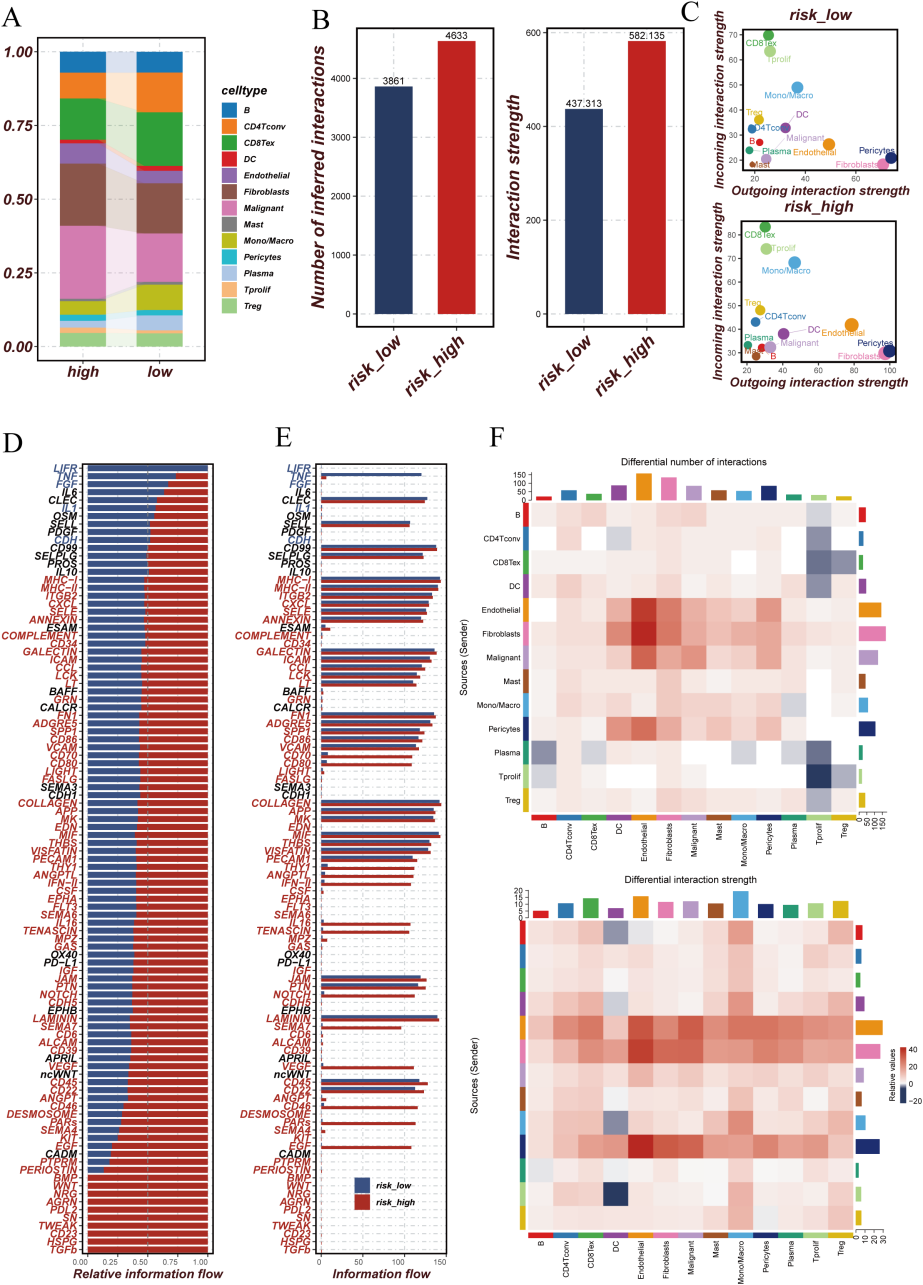
Comparison of cellular interactions and communication patterns between high and low-risk groups. **(A)** Stacked bar plot showing the proportion of different cell types in high and low-risk groups. Each color represents a specific cell type. **(B)** Bar plots comparing the number of inferred interactions and the overall interaction strength between high and low-risk groups. The high-risk group exhibits more interactions and stronger interaction strength compared to the low-risk group. **(C)** Scatter plots showing the outgoing and incoming interaction strength for each cell type in high and low-risk groups. The size of each point reflects the relative interaction strength. **(D)** Bar plot of relative information flow for specific signaling pathways in high and low-risk groups. Pathways with higher flow in the high-risk group are marked in red, while those higher in the low-risk group are in blue. **(E)** Bar plot comparing the absolute information flow for signaling pathways between high and low-risk groups. This highlights the differences in communication intensity across different pathways. **(F)** Heatmaps comparing the differential number of interactions (top) and differential interaction strength (bottom) between high and low-risk groups across different cell types. Red indicates higher values in the high-risk group, while blue indicates higher values in the low-risk group.

Subsequently, we analyzed the expression profiles of DUBGs within the single-cell data, observing distinct expression patterns of these genes across different cell populations (**Figure 6D**). The extracellular matrix is a supportive tissue structure composed of complex molecules such as collagen and fibronectin. It plays a crucial role in normal cell growth and function, while also influencing the infiltration and activity of immune cells within tumors. The extracellular matrix environment surrounding tumor cells can affect the immune response to the tumor, sometimes even forming a barrier that limits the activity of immune cells (32–35). Additionally, there were notable differences in the composition of immune and malignant cells between ESCC patients with high and low-risk scores. High-risk ESCC patients exhibited a higher proportion of malignant cells and fibroblasts, with lower infiltration of CD8 T cells and CD4 T cells compared to those with low-risk scores (**Figure 7A**). Moreover, the number of inferred cell interactions in the high-risk group (4633) was significantly greater than that in the low-risk group (3861) (**Figure 7B**, **Supplementary Figure 3A**). Furthermore, the interaction strength and patterns among certain cell types differed markedly between the high-risk and low-risk groups, with more complex and intense cell-type interactions observed in high-risk patients, which may be associated with their prognosis (**Figure 7C**).

We further identified the cell communication signaling pathways in high-risk and low-risk ESCC patient groups and generated relative information flow diagrams (**Figures 7D, E**) and signal pattern maps (**Figure 7F**). The relative information flow diagrams (**Figures 7D, E**) reveal that the high-risk group exhibits more information flow pathways compared to the low-risk group, with significant enrichment in pathways related to MHC-II, CD34, ICAM, and LIGHT. The signal pattern map (**Figure 7F**, **Supplementary Figure 3B**) shows that the high-risk group has increased interactions among certain cell types, particularly among endothelial cells, fibroblasts, and malignant cells. Moreover, cells in the high-risk group, such as fibroblasts, malignant cells, and macrophages, demonstrate stronger interaction intensities, which may be associated with more complex signaling within the tumor microenvironment. These findings suggest that the tumor microenvironment in high-risk patients is characterized by more active cell communication and more intricate signaling mechanisms.

### DUBGs involved in predicting drug sensitivity in ESCC patients

**Figure 8:**
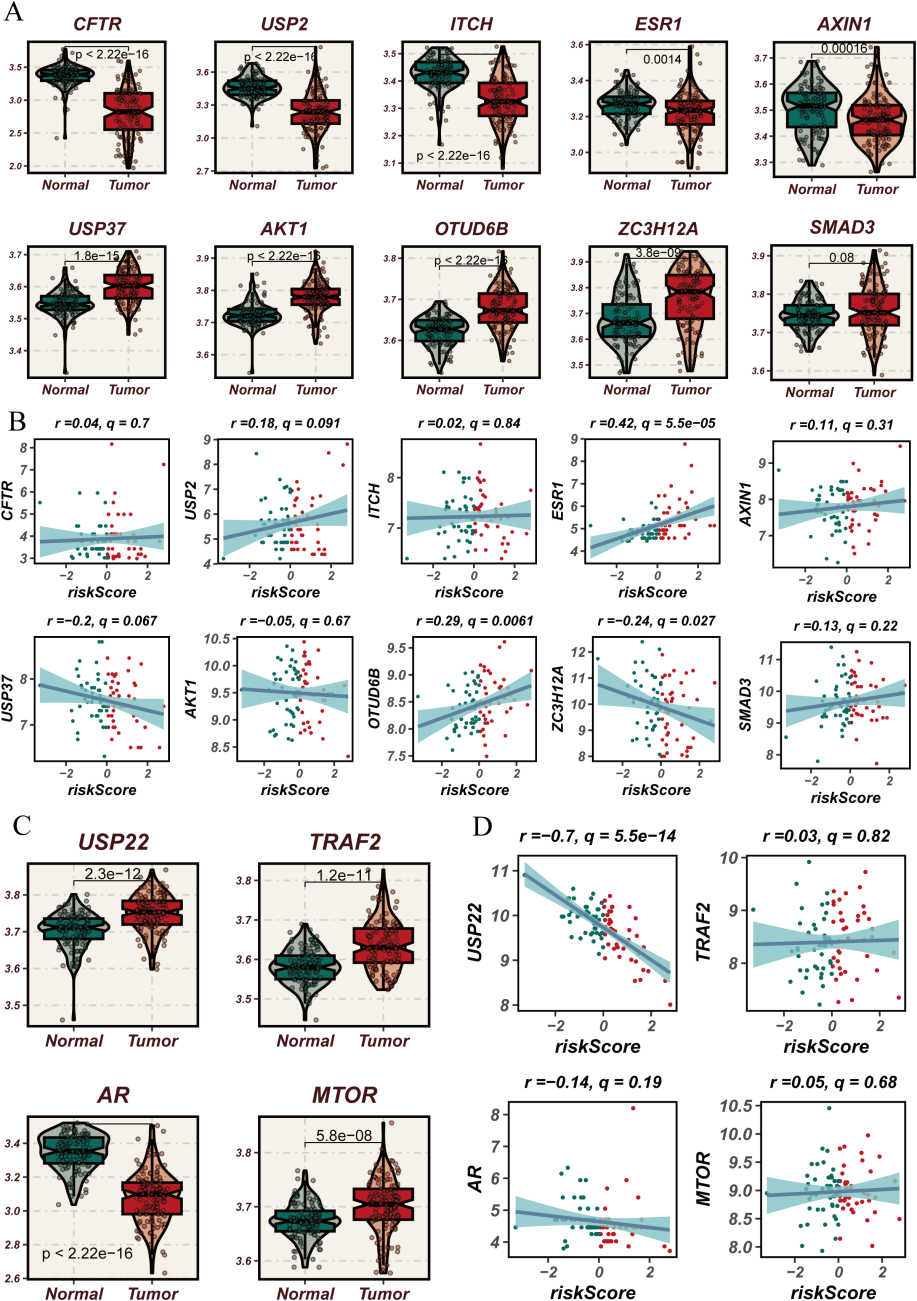
Expression analysis of deubiquitination-related genes used in the modeling process in normal versus esophageal cancer tissues and their correlation with risk scores. **(A)** Violin plots comparing the expression levels of deubiquitination-related genes used in the modeling process (e.g., CFTR, USP2, ITCH, etc.) between normal and esophageal cancer tissues. **(B)** Scatter plots showing the correlation between risk scores and the expression levels of selected deubiquitination-related genes used in the modeling process (e.g., CFTR, USP2, ITCH, etc.) in esophageal cancer. **(C)** Violin plots comparing the expression levels of USP22, TRAF2, AR, and MTOR, which are part of the deubiquitination-related genes used in the modeling process, between normal and esophageal cancer tissues, showing significant differences in expression. **(D)** Scatter plots showing the correlation between risk scores and the expression levels of USP22, TRAF2, AR, and MTOR in esophageal cancer, focusing on the genes used in the modeling process. The blue lines represent the regression lines, and the shaded areas indicate the confidence intervals.

Through the analysis of key DUBGs, we found that genes such as CFTR, USP2, ITCH, ESR1, AXIN1, USP37, AKT1, OTUD6B, ZC3H12A, and SMAD3 are significantly upregulated in tumor tissues compared to normal tissues (**Figures 8A, C**). Additionally, ESR1, OTUD6B, USP2, and USP37 showed a positive correlation with risk scores, while USP22 exhibited a significant negative correlation with risk scores (r = -0.7, p < 0.0001, **Figures 8B, D**), suggesting a potential role for these genes in ESCC tumor progression and patient prognosis. Subsequently, a drug sensitivity analysis revealed differences in the sensitivity to certain common drugs between high-risk and low-risk groups. Drugs such as BMS-536924, BDP-00009066, AUZ-12345, and AZ6102 showed significantly higher response levels in the high-risk group compared to the low-risk group, indicating the potential therapeutic advantages of these drugs in high-risk patients (**Supplementary Figure 2A**).

### MTOR^+^ tumor cells may serve as a therapeutic target for ESCC patients

We incorporated a total of 14 DUBGs (USP2, ITCH, ESR1, AXIN1, MTOR, USP22, TRAF2, USP37, AKT1, AR, OTUD6B, ZC3H12A, and SMAD3) into our risk model. Among these, the MTOR gene exhibited a higher Cox coefficient in our prognostic model. Mammalian target of rapamycin (mTOR), the final component of the PI3K/mTOR pathway, plays a crucial role in regulating cell proliferation, growth, metabolism, and protein synthesis (36). Previous studies have highlighted the significant role of the MTOR gene in tumorigenesis and cancer progression. Mutations in the MTOR gene can result in the persistent hyperactivation of the mTOR signaling pathway, and over 30 mTOR gene mutations have been identified across various cancer types (37). These mutations are not only associated with enhanced mTOR protein activity but have also been linked to resistance mechanisms against mTOR inhibitors (38).

**Figure 9:**
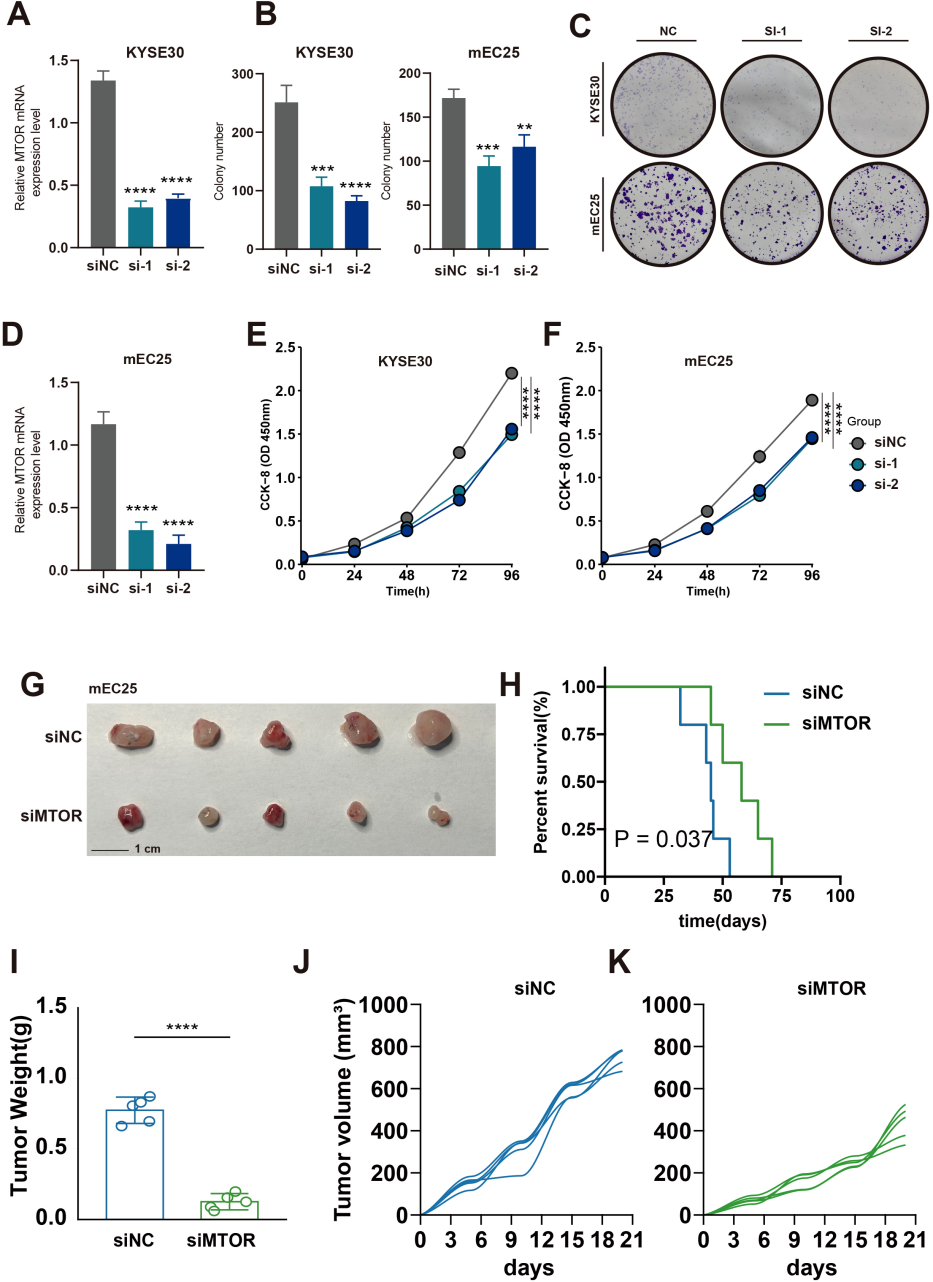
Effect of MTOR Knockdown on Cell Growth, Colony Formation, and Tumorigenicity in Esophageal Cancer Cells. **(A, D)** Relative mRNA expression levels of MTOR in KYSE30 **(A)** and mEC25 **(D)** cells following siRNA-mediated knockdown (si-1, si-2) compared to the negative control (siNC). A significant reduction in mRNA levels was observed in the siRNA-treated groups. ****p < 0.0001. **(B, C)** Quantification and representative images of colony formation assays in KYSE30 and TE1 cells **(B)** and KYSE30 and mEC25 cells **(C)** after siRNA-mediated knockdown of MTOR (si-1, si-2) compared to the negative control (NC). The number of colonies was significantly reduced in the siRNA-treated groups. ***p < 0.001, **p < 0.01. **(E)** Cell proliferation assay (CCK-8) in KYSE30 cells showing reduced cell growth in the siMTOR groups (si-1, si-2) compared to the negative control (siNC). **(F)** Cell proliferation assay (CCK-8) in mEC25 cells showing reduced cell growth in the siMTOR groups (si-1, si-2) compared to the negative control (siNC). **(G)** Representative images of tumors collected from mice injected with mEC25 cells transfected with either negative control siRNA (siNC) or siRNA targeting the gene (siMTOR). The si3 group showed smaller tumors. **(H)** Kaplan-Meier survival curve of mice injected with mEC25 cells. Mice in the siMTOR group had significantly longer survival times compared to the siNC group (P = 0.037). **(I)** Tumor weight comparison between the siNC and siMTOR groups. Tumors from the siMTOR group were significantly lighter than those from the siNC group. ****p < 0.0001. **(J, K)** Tumor volume growth curves for the siNC group **(J)** and the siMTOR group **(K)**, showing that the tumors in the siMTOR group grew more slowly compared to those in the siNC group.

To investigate the role of MTOR in esophageal cancer, we constructed MTOR knockdown cell lines, KYSE30 and mEC25, using siRNA technology. Five days post-transfection, qRT-PCR was performed to assess MTOR expression levels, confirming the effectiveness of MTOR knockdown in the KYSE30 and mEC25 cell lines (**Figures 9A, D**). Functional assays revealed that MTOR knockdown significantly reduced colony formation efficiency and cell proliferation in esophageal cancer cells (**Figures 9B, C, E, F**). Additionally, *in vivo* experiments using a subcutaneous tumor model in mice, established with the mEC25 cell line, demonstrated that MTOR knockdown slowed tumor growth and prolonged survival (**Figure 9G–K**). These findings strongly suggest that the MTOR gene is a risk factor for esophageal squamous cell carcinoma (ESCC). Further research is warranted to develop more effective diagnostic and therapeutic strategies for ESCC patients.

## Discussion

Esophageal cancer is one of the most heterogeneous, common, and deadly types of cancer, particularly in East Asia (8, 39). However, compared to other common tumor types, research on esophageal cancer is notably lacking, and there has been limited progress in treatment over the past few decades (40, 41). Deubiquitination is a process catalyzed by DUBs that removes ubiquitin from ubiquitinated proteins, effectively reversing the ubiquitination process. The dynamic interplay between ubiquitination and deubiquitination is closely linked to various cellular functions, and its dysregulation can lead to a range of diseases, including neurodegenerative disorders and cancer (19). Recent studies suggest that deubiquitination can affect the MTOR pathway, a crucial regulator in cell growth and metabolism (Zhao et al., 2021). For instance, the enzyme USP39 promotes mTORC2 activation, further enhancing tumor progression (42). Additionally, inhibitors targeting DUBs like USP14 and UCH37 show potential for controlling MTOR pathway activation, highlighting their therapeutic relevance (Sha et al., 2019) (43). Understanding these processes could offer new insights into cancer diagnosis and treatment. In this study, we investigated the expression and significance of DUBGs in ESCC, revealing their prognostic value. Moreover, through *in vitro* cell experiments and *in vivo* subcutaneous tumor models in mice, we validated that the knockdown of the key deubiquitination-related gene MTOR significantly inhibits the malignant biological behavior of esophageal cancer cells, suggesting its potential as a therapeutic target for esophageal cancer patients.

ESCC is considered a heterogeneous group of cancers, and this study provides a comprehensive analysis of the biological processes in ESCC patients influenced by DRGS. By stratifying ESCC patients into high-risk and low-risk groups based on the median DRGS score, we demonstrated that DRGS serve as reliable predictors of patient prognosis. High-risk ESCC patients consistently exhibited poorer survival outcomes compared to their low-risk counterparts. Furthermore, integrating DRGS stratification with TMB analysis provided additional insights into patient outcomes. Specifically, patients categorized in the high-risk and high-TMB groups exhibited markedly reduced survival probabilities, highlighting a synergistic impact of DRGS activity and high TMB on patient prognosis. These findings underscore the utility of DRGS in predicting survival outcomes and emphasize their potential to inform risk-adapted therapeutic strategies in ESCC management (44). Differences between high-risk and low-risk groups were also observed in TMB, ESTIMATE score, immune score, and tumor purity. Specifically, high-risk patients tended to exhibit higher TMB and altered ESTIMATE scores, reflecting significant variations in immune and stromal components within the tumor microenvironment. The tumor immune microenvironment encompasses a complex network of interactions between tumor cells and their surroundings, including immune cells, inflammatory cells, blood vessels, and the extracellular matrix. These components collectively play a pivotal role in modulating tumor progression, immune evasion, and therapeutic response. High-risk ESCC patients, characterized by distinct immune microenvironment profiles, may thus demonstrate varying degrees of sensitivity to immunotherapy and other targeted treatment modalities, emphasizing the need for personalized therapeutic strategies tailored to tumor microenvironment characteristics (45–47). Specifically, high-risk patients showed increased infiltration of malignant cells and fibroblasts but decreased infiltration of immune cells such as CD8+ T cells and CD4+ T cells, suggesting that DRGS may influence the tumor immune microenvironment in ESCC patients, thereby impacting their prognosis.

To further explore the impact of DRGS on the microenvironment and potential biological processes in ESCC patients, we integrated single-cell analysis for additional validation. The analysis revealed that high-risk ESCC patients exhibit significantly higher levels of cellular interaction frequency, interaction strength, and network complexity compared to low-risk patients, suggesting a highly intricate signaling network within the tumor microenvironment. These complex interactions might involve enhanced crosstalk between tumor cells, immune cells, and stromal components, potentially contributing to the aggressiveness and therapy resistance in high-risk patients. Furthermore, DRGS appear to serve as reliable predictors of drug sensitivity, as high-risk ESCC patients demonstrated significantly greater responsiveness to targeted therapeutics such as BMS-536924, BDP-00009066, AUZ-12345, and AZ6102. These results highlight the potential for DRGS-based stratification to guide precision oncology approaches, enabling the development of more effective and personalized treatment strategies.

The hyperactivation of the MTOR pathway is increasingly recognized as a driver of tumor recurrence and drug resistance across multiple cancer types, including ESCC (48). Our findings align with these observations, demonstrating that MTOR plays a central role in enabling metabolic adaptations that support rapid tumor growth. Specifically, MTORC1 activation drives glycolysis, producing glycolytic intermediates essential for synthesizing macromolecules like proteins, lipids, and nucleotides. Concurrently, mTORC2 activation enhances mitochondrial ATP production, supporting the high bioenergetic demands of proliferating tumor cells. Furthermore, our data suggest that MTOR activation promotes glutamine uptake and lipid oxidation, fueling the tricarboxylic acid (TCA) cycle to sustain mitochondrial function. Notably, knockdown of MTOR in esophageal cancer cell lines significantly inhibited cell proliferation and colony formation, while *in vivo* experiments demonstrated reduced tumor growth rates and prolonged survival in mouse models. These findings underscore the potential of targeting MTOR as a therapeutic strategy in ESCC. Moreover, given MTOR’s pivotal role in nutrient sensing and metabolic reprogramming, its inhibition may simultaneously reduce tumor resilience to therapeutic interventions and sensitize tumors to existing treatment modalities, ultimately improving patient outcomes.

Proliferating cancer cells require increased synthesis of proteins, lipids, and nucleotides (49). Glycolysis can be upregulated through the activation of mTORC1, providing more glycolytic intermediates for the biosynthesis of these macromolecules (50, 51). Additionally, the activation of mTORC1 promotes the uptake of glutamine to sustain mitochondrial ATP production. Fatty acids can also supply carbon to the tricarboxylic acid (TCA) cycle, thereby supporting mitochondrial function (52, 53). MTOR, as one of the key genes in DUBGs, plays a crucial role in these processes. Through knockdown of the MTOR gene in esophageal cancer cells, we found that reducing MTOR expression significantly inhibits tumor cell proliferation and colony formation, suppresses tumor growth rate in a mouse subcutaneous tumor model, and prolongs survival in mice. These findings suggest that MTOR could be a potential therapeutic target for ESCC patients.

## Conclusion

In this study, we developed a predictive risk scoring model based on DRGS that effectively forecasts patient prognosis and is closely associated with tumor mutational burden, immune cell infiltration, tumor purity, and drug sensitivity, highlighting its clinical relevance. Additionally, MTOR has been identified as a potential therapeutic target in esophageal cancer, underscoring its critical role in tumor progression. These findings enhance our understanding of the pathogenesis of esophageal cancer and offer new insights for the development of personalized treatment strategies.

## Data Availability

The original contributions presented in the study are included in the article/**Supplementary Material**. Further inquiries can be directed to the corresponding author.

## Supplementary material

Supplementary Figure 1 Survival analysis and ROC curves of esophageal cancer patients based on risk scores derived from deubiquitination-related genes. **(A)** Kaplan-Meier survival curves comparing overall survival between high-risk and low-risk groups of esophageal cancer patients. The high-risk group shows significantly poorer survival compared to the low-risk group (P < 0.0001).

Supplementary Figure 2 Drug sensitivity analysis in esophageal cancer based on risk groups. **(A)** Violin plots comparing the sensitivity of esophageal cancer cells to various drugs (e.g., BMS-538924, BDP-00009086, etc.) between high-risk and low-risk groups. The sensitivity is measured as the drug response score, with higher scores indicating greater sensitivity. The p-values indicate the statistical significance of the differences between the two groups.

Supplementary Figure 3 Cell-cell interaction networks and signaling patterns in low-risk and high-risk groups in esophageal cancer. **(A)** Network diagrams illustrating the number of cell-cell interactions in the low-risk (left) and high-risk (right) groups. Each node represents a different cell type, and the thickness of the lines indicates the number of interactions between cell types. The high-risk group shows an increased number of interactions compared to the low-risk group. **(B)** Heatmaps showing the outgoing signaling patterns in low-risk (left) and high-risk (right) groups. The heatmaps compare the relative strength of signaling pathways between different cell types. The high-risk group exhibits more robust and diverse signaling interactions.
