# Supplementary figures and images for: Leveraging single-cell and multi-omics approaches to identify MTOR-centered deubiquitination signatures in esophageal cancer therapy
Source: Front Immunol. 2024 Dec 17;15:1490623. doi: 10.3389/fimmu.2024.1490623 (PMC11685190; doi:10.3389/fimmu.2024.1490623)

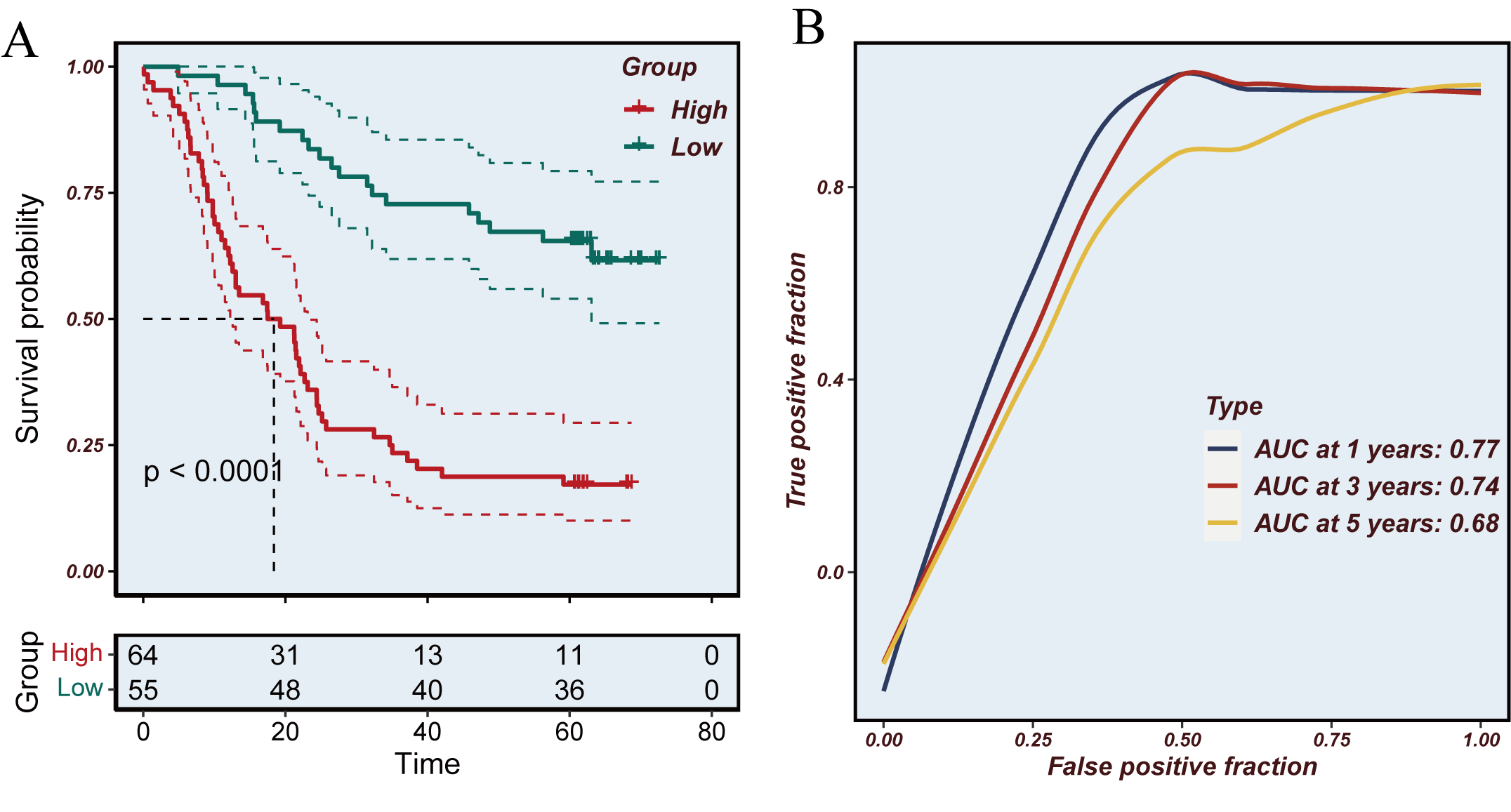

Supplement: Supplementary Figure 1 — Survival analysis and ROC curves of esophageal cancer patients based on risk scores derived from deubiquitination-related genes. (A) Kaplan-Meier survival curves comparing overall survival between high-risk and low-risk groups of esophageal cancer patients. The high-risk group shows significantly poorer survival compared to the low-risk group (P < 0.0001). [file Image1.tif]

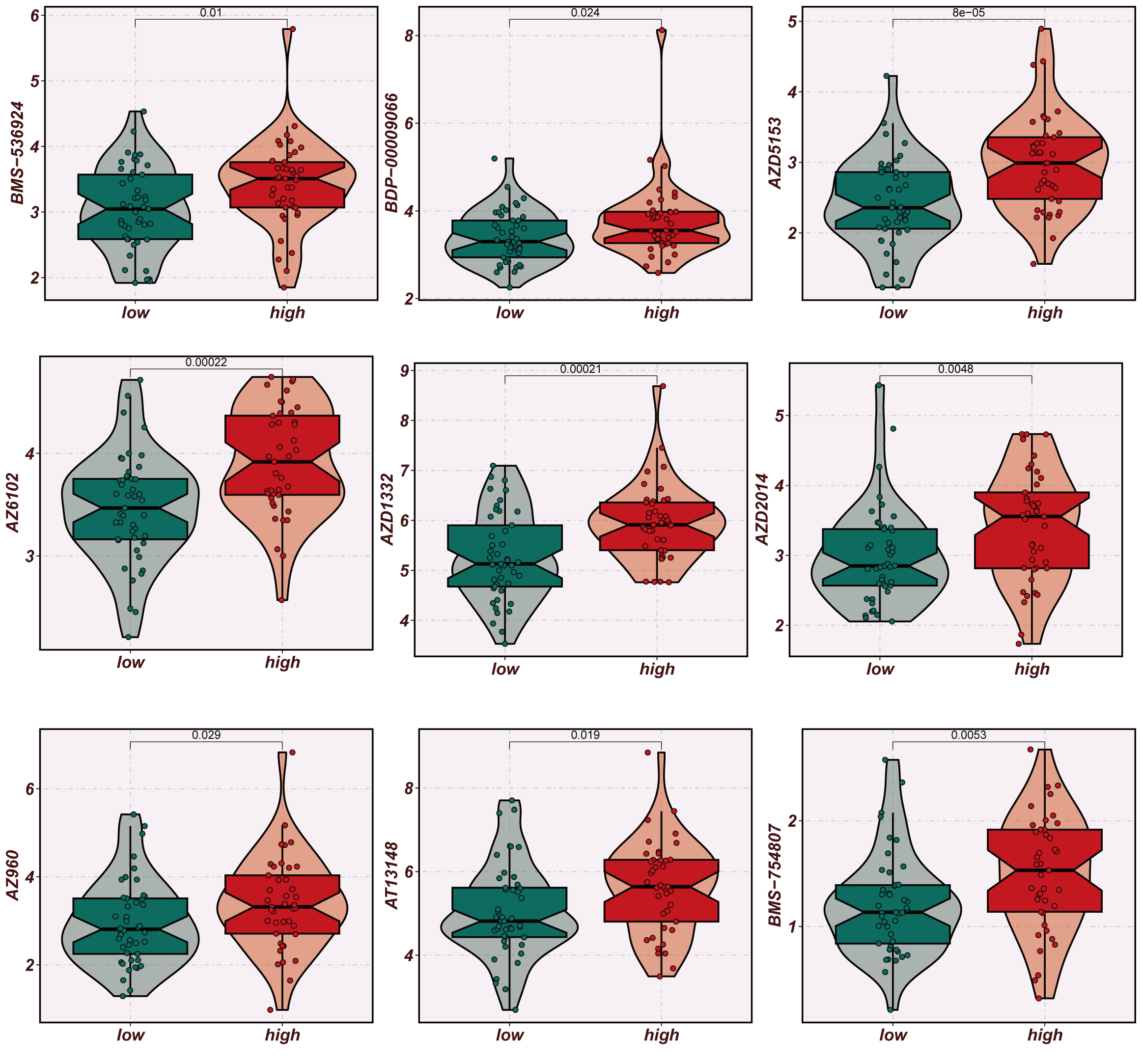

Supplement: Supplementary Figure 2 — Drug sensitivity analysis in esophageal cancer based on risk groups. (A) Violin plots comparing the sensitivity of esophageal cancer cells to various drugs (e.g., BMS-538924, BDP-00009086, etc.) between high-risk and low-risk groups. The sensitivity is measured as the drug response score, with higher scores indicating greater sensitivity. The p-values indicate the statistical significance of the differences between the two groups. [file Image2.tif]

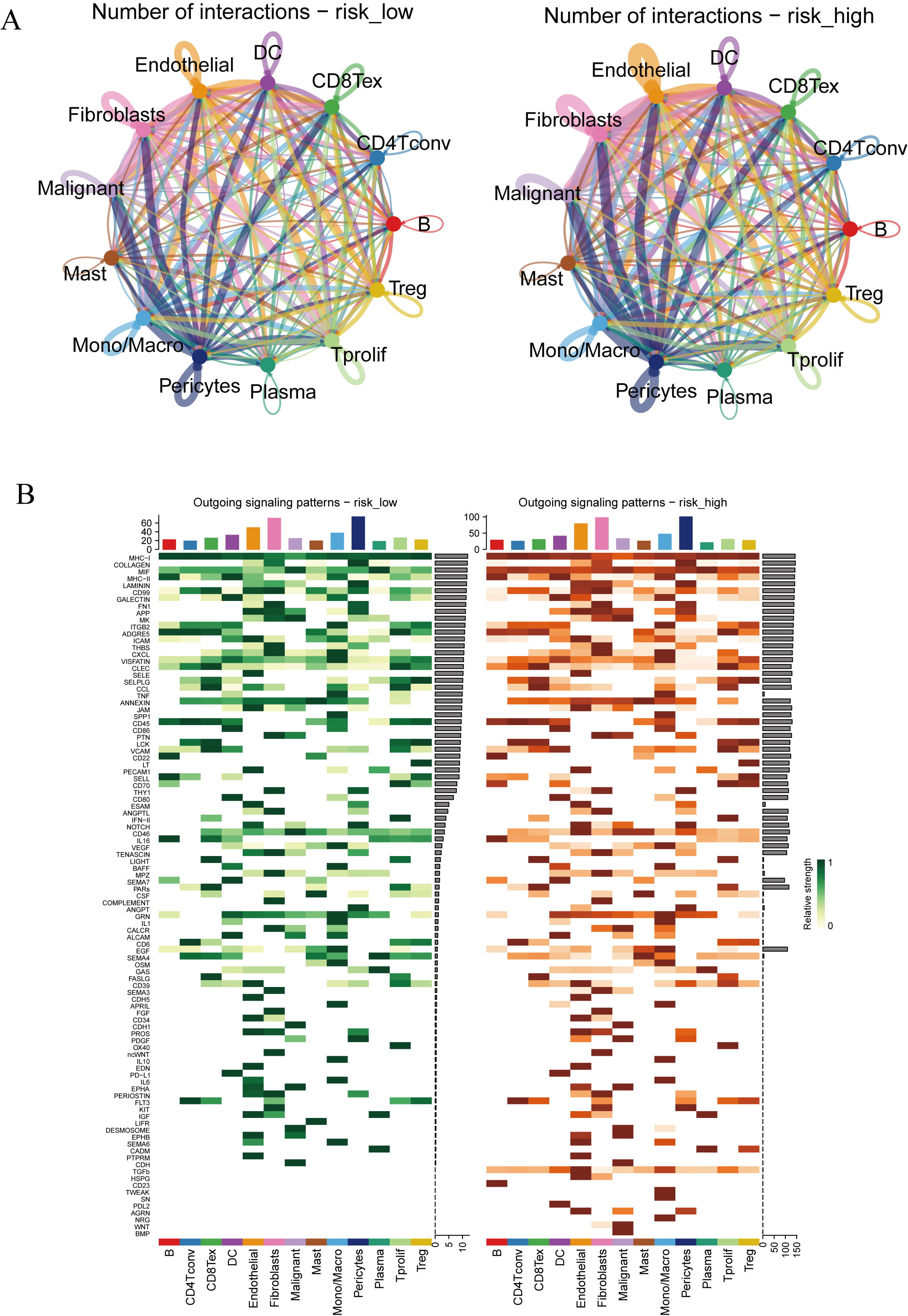

Supplement: Supplementary Figure 3 — Cell-cell interaction networks and signaling patterns in low-risk and high-risk groups in esophageal cancer. (A) Network diagrams illustrating the number of cell-cell interactions in the low-risk (left) and high-risk (right) groups. Each node represents a different cell type, and the thickness of the lines indicates the number of interactions between cell types. The high-risk group shows an increased number of interactions compared to the low-risk group. (B) Heatmaps showing the outgoing signaling patterns in low-risk (left) and high-risk (right) groups. The heatmaps compare the relative strength of signaling pathways between different cell types. The high-risk group exhibits more robust and diverse signaling interactions. [file Image3.tif]
